# Endoplasmic Reticulum Chaperones and Their Roles in the Immunogenicity of Cancer Vaccines

**DOI:** 10.3389/fonc.2014.00379

**Published:** 2015-01-06

**Authors:** Michael W. Graner, Kevin O. Lillehei, Emmanuel Katsanis

**Affiliations:** ^1^Department of Neurosurgery, Anschutz Medical Campus, University of Colorado School of Medicine, Aurora, CO, USA; ^2^Department of Pediatrics, The University of Arizona, Tucson, AZ, USA

**Keywords:** endoplasmic reticulum, cancer vaccine, chaperones, CRCL, immunotherapy

## Abstract

The endoplasmic reticulum (ER) is a major site of passage for proteins en route to other organelles, to the cell surface, and to the extracellular space. It is also the transport route for peptides generated in the cytosol by the proteasome into the ER for loading onto major histocompatibility complex class I (MHC I) molecules for eventual antigen presentation at the cell surface. Chaperones within the ER are critical for many of these processes; however, outside the ER certain of those chaperones may play important and direct roles in immune responses. In some cases, particular ER chaperones have been utilized as vaccines against tumors or infectious disease pathogens when purified from tumor tissue or recombinantly generated and loaded with antigen. In other cases, the cell surface location of ER chaperones has implications for immune responses as well as possible tumor resistance. We have produced heat-shock protein/chaperone protein-based cancer vaccines called “chaperone-rich cell lysate” (CRCL) that are conglomerates of chaperones enriched from solid tumors by an isoelectric focusing technique. These preparations have been effective against numerous murine tumors, as well as in a canine with an advanced lung carcinoma treated with autologous CRCL. We also published extensive proteomic analyses of CRCL prepared from human surgically resected tumor samples. Of note, these preparations contained at least 10 ER chaperones and a number of other residents, along with many other chaperones/heat-shock proteins. Gene ontology and network analyses utilizing these proteins essentially recapitulate the antigen presentation pathways and interconnections. In conjunction with our current knowledge of cell surface/extracellular ER chaperones, these data collectively suggest that a systems-level view may provide insight into the potent immune stimulatory activities of CRCL with an emphasis on the roles of ER components in those processes.

## Introduction

The endoplasmic reticulum (ER) is an organelle of new beginnings, sudden endings, twists, turns and connections, major changes, and passage to new places. During protein translation, nascent proteins destined for the ER or other locations along the secretory route protrude an appropriate “signal sequence” from the ribosome that the signal recognition particle (SRP) distinguishes as an ER address label (1). After the SRP binds to the peptide, it tethers the ribosome near the SRP receptor on the ER membrane. The ribosome docks with the SEC61 complex for co-translation of the rest of the protein across the ER membrane (2, 3). Once in the ER, chaperone-based folding occurs, along with glycosylation, disulfide bond formation, and transport out of the ER into the Golgi if such address labels are found in the newly minted protein (4). These activities require calcium, and the ER (along with mitochondria) is the major calcium storage compartment in a typical cell. Many of the chaperones are calcium-binding proteins with extensive capacity; this plays into their functions, as well as to other calcium-essential units in the cell (5). The oxidizing environment of the ER lumen promotes disulfide bridge formation, largely via protein disulfide isomerase (PDI/PDIA) family members, and these bonds are likely critical in the proper folding of individual proteins and in formation of multi-subunit complexes (6). The ER has numerous quality control (ERQC) mechanisms to assure properly folded proteins exit the ER for other destinations, but may essentially end in ER-associated degradation (ERAD) (7). Proteins that do not achieve the appropriate tertiary or quaternary confirmations are considered terminally misfolded and are poly-ubiquitinated (in a complex fashion) with retrotranslocation to the cytosol for proteasomal degradation (8). The efficiency of entry, exit, and arrival at the final destination varies dramatically for different proteins and ranges from nearly 100% “success” (i.e., amount of a given protein entering the ER compared to the amount of that protein reaching its final localization, such as the cell surface) to as low as 25% (9). The lumenal environment of the ER is most akin to the cell’s exterior, and the ER is a portal connecting the cytosol to the cell surface and beyond.

The chaperones of the ER are critical to many aspects of ER function, whether in protein folding modes, as calcium binders, as sensors of stress such as the unfolded protein response (10), or due to cell-surface localization or extracellular release, as immune modulators (11–18). These latter characteristics combine with the protein- and peptide-binding/carrying capacity of chaperones to allow for their utilization as vaccines, particularly in oncology (19–22). This review will highlight the multifaceted roles of the ER in immunity, and will then focus on how chaperones from the ER may contribute to immune responses under “exogenous” circumstances, e.g., once outside the cell. We will further discuss how such chaperones may contribute to anti-cancer immunity in a complex vaccine like chaperone-rich cell lysate (CRCL). As we will discuss a number of chaperone proteins from various subcellular locations beyond those of the ER, we have prepared Table 1 to aid in keeping track of these proteins.

**Table 1:** **Chaperone proteins described herein and their subcellular localizations**.

		Subcellular localization					
Protein common name	Gene name	Endoplasmic reticulum/Golgi	Cytosol	Nucleus	Mitochondria	Lysosome	Cell surface^a^
HSP27^b^	HSPB1		X	X			X
HSP47 (serpin H1)	SERPINH1	X					X
HSP60	HSPD1		X		X		X
HSP70	HSPA1A/B		X	X		X	X
HSC70	HSPA8		X	X		X	X
GRP78 (BiP)	HSPA5	X	X	X	X		X
HSP90	HSP90AA/B1		X	X			X
HSP110	HSPH1		X	X			X
GRP94 (gp96)	HSP90B1	X		X			X
GRP170 (ORP150)	HYOU1	X					X
PDI/PDIA^c^	P4HB	X					X
CRT/CALR	CALR	X	X				X

*Chaperones in this article and their subcellular localizations. This is a list of the chaperone proteins and their gene names mentioned in this article annotating their known subcellular localizations*.

*^a^Cell-surface localization is most often associated with tumor cell surfaces*.

*^b^Murine version is often called HSP25*.

*^c^There are muliple PDI (protein disulfide isomerase) family members too numerous to include here*.

## The Endoplasmic Reticulum as a Conduit to Immunity: T Cells “See into the Soul” of a Cell

The mammalian immune system has developed a largely “non-invasive” means of assessing the immune status of most of the host organism’s cells. Immune effector cells of both the adaptive arm (i.e., CD4^+^ and CD8^+^ T cells) and the innate arm [e.g., natural killer (NK) cells] monitor cell surfaces by engaging major histocompatibility complex class I and II (MHC I and II) molecules in the case of T cells (23–25) and damage-associated molecular patterns (DAMPs) (26), as well as stress ligands such as MICA/B and ULBP families (27) in the case of NK cells. NK cells also balance activating and inhibitory receptor stimulation that may be present on normal cells, or downregulated or absent on abnormal cells, such as loss of MHC I (28). Perturbations that occur in the cytosol such as pathogenic infection or the genetic, proteomic, and metabolic disarray of neoplasia may lead to the expression of non-self proteins or of mutated self proteins. These, along with other “normal” but obsolete proteins are poly-ubiquitinated and are targeted for degradation into short peptides by the proteasome. With additional trimming (or outright proteasome-independent generation) possible by cytosolic peptidases, peptides enter the ER through the TAP transporters (transporters associated with antigen processing; ABC family members). There, the peptides may be further pruned before chaperone-assisted loading onto MHC I molecules, which are then packaged for transit to the cell surface. Display of peptides in the context of MHC I molecules provides the reading frame for CD8^+^ T cells that determine the normal or abnormal status of the presenting cell.

MHC II display and presentation generally only occurs in specialized immune cells known as professional antigen-presenting cells (APCs), such as macrophage, dendritic cells (DCs), and B cells (29). However, MHC II expression can occur on other cells such as endothelial cells following IFNγ exposure (30) or on neuronal cells in peripheral neuropathies (31), turning such cells into APCs. Exogenous antigens are engulfed at the cell surface into endosomal/phagosomal vesicles (32) where denaturation and degradation of proteins begins. Meanwhile, MHC II molecules are assembled in the ER with a “placeholder” in the peptide-binding cleft, the chaperone invariant chain (Ii). The MHC II molecules enter vesicles and are released into the cytosol’s endocytic pathway where the Ii is cleaved to class II-associated invariant chain peptide (CLIP). These vesicles encounter the late endocytic/phagocytic vesicles with lysosomal characteristics where CLIP is displaced by peptides with higher affinity for the MHC binding pocket. These are often called MHC II compartments (MIICs), and the vesicles eventually deliver MHC II to the cell surface for presentation to CD4^+^ T cells (33) for the latters’ assessments of the immune status of the presenting cell. Recent work with the MHC II process describes more complicated routes and alternatives, and some of this information will re-appear in our discussion of antigen cross-presentation (34, 35).

Thus, CD4^+^ and CD8^+^ T cells scan cell surfaces for the MHC-displayed peptides that may indicate a pathologic state within those cells; however, the T cells require activation and “education” concerning the nature of the problem. Professional APCs serving as scavenger cells may have confronted a situation with cell/tissue damage that resulted in the APCs engulfing extracellular material. If this occurred in an inflammatory environment in the presence of released “danger signals” (36, 37), the APCs become stimulated to provide “signal one” and “signal two” to T cells (38, 39). The first signal is the MHC-restricted peptide that is capable of triggering a T-cell receptor (TCR) specific for that particular peptide in that particular MHC peptide-binding cleft; the assumption is that the peptide is indicative of the distress (infection, mutation) in the donor cell before acquisition by the APC. The second signal comes from the expression of co-stimulatory molecules by the APC, such as CD80/CD86, that provide activation impetus for T cell. The interface between APC and T cell in this scenario is called the “immunological synapse” (40). After recognition of antigen, activation, and stimulation, the T cells exit the lymph node and enter the periphery to search for affected cells that display the antigens that indicate disease (i.e., the same ones that triggered the TCRs originally).

In the scenario described, APCs acquire exogenous antigens that are displayed to T cells; the classical mode of antigen uptake and display by MHC in this trafficking pathway is via MHC II, which would induce only a CD4^+^ T-cell response. However, it is clear that APCs also display foreign and self peptides on MHC I molecules, called “cross-presentation” (41). The endosomal trafficking of endocytosed proteins, particularly in professional APCs, can direct such vesicles away from lysosomal degradation; peptide generation within endosomes may allow for direct loading of vesicle-bound MHC I molecules (42). On the other hand, the proteins or peptides could passage out of the vesicles and into the cytosol for proteasomal processing and entry into the classical MHC I pathway. These peptides could also passage back into endosomes via endosomal TAP transporters; if MHC I molecules are in those vesicles, the peptides may be loaded onto the presentation proteins (42).

Through these various mechanisms, which start with the assembly of MHC molecules in the ER, T cells may be stimulated to respond to a pathogenic state, and during surveillance are able to determine the internal stasis or possible malcontented nature of the MHC-presenting cell. The “outside-looking-in” format does not require destruction of the presenting cell, unless that cell displays antigens indicative of a pathogenic state.

## Chaperones on the Roof: Cell-Surface Chaperones in Immunity

Roles of chaperones in the antigenicity of foreign, and possibly self peptides and proteins, are usually associated with extracellular chaperones as danger signals (36, 37, 43–45). The strong sequence and structural relationships between chaperones from primitive and more advanced organisms (46) suggest that immune reactivity that evolved against bacterial chaperones may lead to cross-reactivity with mammalian chaperones [e.g., Ref. (47)], but those outcomes are varied (48). Binding to pattern recognition receptors such as Toll-like receptors (TLRs) is a characteristic of extracellular chaperones, both mammalian and bacterial (49, 50) but TLR stimulation may, at least in some cases, result from bacterial PAMPs associated with the chaperones (51). Nonetheless, the innate immune signaling aspects of chaperones outside the cell are likely the key initial mediator steps in promoting an immune response.

Cell-surface display of chaperones represents a special case of re-localized chaperones capable of provoking immune responses. In oncology, membrane HSP70 is one of the most heavily studied (52), where a 14-mer region of the chaperone is recognized as a target for NK cells (53). The mechanism for the HSP70’s membrane association remains unclear, although its interactions with negatively charged phospholipids may play a role (54), with involvement of particular domains of the protein (55). The “large” relative of HSP70, HSP110 (22), has been noted on the surfaces of brain tumor cell lines (15, 16), but the implications of this localization are unknown.

The small heat-shock protein HSP27 (HSP25 in mice) was one of the number of chaperone proteins found on tumor cell surfaces by proteomic analyses (56), as well as by flow cytometry (15). The immune responses to surface HSP27 remain unclear, but murine mammary cancer cells selected for cell-surface expression of HSP25 proliferated faster and exhibited more frequent lung metastatic lesions than cells with lower or minimal surface HSP25 display (57). Interestingly, in those immune-competent animals, heat-shock-driven inducible HSP70 surface expression on those cells resulted in reduced metastatic growth and overall increased survival compared to implantation of cells with low surface HSP70 expression, suggesting that immune responses may play a role, perhaps via NK cells (53).

HSP90 was one of the original chaperones found to be a “tumor-specific transplantation antigen” (TSTA) potentially useful as a vaccine when purified from tumors (58), and was shown to be present on murine MethA tumor cell surfaces. The surface expression was discovered on other tumor cell lines as well (59). Surface HSP90 interacts with HER2 and mediates tumor cell invasiveness on breast cancer cells (60), and blocking surface HSP90 activity with a cell-impermeant inhibitor or antibodies validates this in other tumor types (61–63). The chaperone was also identified on the surfaces of CNS/neuronal-derived tumors (15, 64), where expression on spheroid lines was higher (64). Since the growth of CNS tumors in “stem cell-like” cultures (that frequently form spheroids) is a relatively new phenomenon, HSP90 surface expression may need to be re-examined for those tumors. As mentioned above, tumor-surface HSP70 is a known NK cell target, but both surface HSP70 and HSP90 are also gamma–delta T-cell targets, at least in EBV-transformed B cells (65, 66).

While the mechanisms of cell-surface display for chaperones considered to be canonically localized to the cytosol (or nucleus, in some cases) remain puzzling, one can imagine a simpler route to the cell surface for chaperones originally localized to the ER. As they are residents of the compartment of origin for proteins destined for cell-surface expression or extracellular release, their passage out of the ER requires bypassing KDEL receptors. These are proteins in pre- or *cis*-Golgi compartments that recognize the lys–asp–glu–leu (KDEL) motif present on most ER resident proteins. Those proteins that progress from the ER into the Golgi compartments are recognized and bound by the family of KDEL receptors that then engage in retrograde transport to return the KDEL-containing proteins to the ER (67).

Of the cell surface-expressed ER chaperones, GRP78 (BiP) is one of the best characterized and was noted on the surfaces of a hybrid neuroblastoma cell line in the late 1990s (68) (and has been found on other CNS/neurologic tumors) (15, 16). GRP78 was also one of the chaperone proteins identified on tumor cell surfaces in a proteomic study (56) (along with other HSP70 family members, and HSPs 27, 47, and 60, and PDI members). GRP78’s chaperone capacity, apparently still intact on the cell surface, was used to target pro-apoptotic peptides fused to consensus GRP78 binding motifs resulting in cell death and reduced model tumor growth (69) [and further reviewed here (70)]. At the cell surface, GRP78 acts as a receptor or in complexes with numerous partners that may promote cell survival or engage in apoptosis (71). Surface GRP78 is a therapeutic antibody target (72, 73), but in some cases antibodies in patient sera bind to activated α2-macroglobulin’s agonist site on its receptor GRP78. This leads to tumor cell growth stimulation and apoptosis prevention (74). In general, tumor-surface GRP78 is indicative of enhanced malignant tumor phenotypes (71).

GRP94 (also called gp96, endoplasmin, ERp99), is the ER HSP90 paralog. Like HSP90, it was identified as a “tumor rejection antigen” (TRA) purified from MethA and CMS5 murine sarcomas (75), and was found in plasma membrane fractions and on murine and human tumor cell surfaces (76–78). The function of surface GRP94 is not entirely clear, but it appears to play a role in the processing of surface metalloproteinases (79). Immunologically, surface GRP94 can activate DCs, inducing a pro-inflammatory state with activation of tumor-specific T cells (80).

Despite its prominent role as an ER chaperone cancer vaccine (81), GRP170/ORP150 has only rarely been cited as a cancer cell-surface protein (15, 82), but it has been identified on mouse egg oolemma (83) as well as human sperm surfaces (84, 85). Other ER residents such as ERp5/PDI6 (PDI family) are present on tumor cell surfaces; in this case, the chaperone is involved in the release of the NK cell activating receptor MICA from tumor cell surfaces, presumably as a protective measure to avoid NK attack. PDI also functions in the shedding of tumor endothelial maker 5 (TEM5) with potential impacts on cell adhesion and migration (86). PDI and calreticulin were among the KDEL-containing proteins previously identified as surface components of the NG108-15 cell line (68). PDI family members had been identified as localized to platelet surfaces as early as 1995 (87) and were later shown to be on B-CLL cells (88). PDI plays a role in glioma xenograft tumor invasiveness (89). Roles for surface PDIs include transnitrosation and nitric oxide metabolism (86) and formation of thiols on cell-surface proteins (90).

Calreticulin (CRT; CALR) is considered as an ER chaperone, but with very divergent intracellular, cell-surface, and extracellular localizations (91). It was identified with cell surfaces as early as 1995 (92, 93), and is regarded as a major immunologic player whose surface exposure promotes the immunogenicity of tumor cells dying by particular chemotherapy agents (94). CRT was already known as a tumor peptide-carrying cancer vaccine candidate (95–97), but in these scenarios of (normally immune-silent) apoptotic cell death, it is viewed as an engulfment signal for phagocytic cells such as macrophage and DCs (98, 99). While CRT is clearly present on numerous cell types, including cancers (15, 91), those cells may resist APC interactions and phagocytosis via CD47 (98, 100). These studies strongly suggest that how tumor cells die matters greatly to the immune system, and delineate potential avenues of improved therapy.

Thus, cell-surface localization of various chaperone proteins, while originally quite controversial, is now accepted, and seems to associate with cancer pathology. The roles of surface chaperones in anti-tumor immunity may be complicated in terms of putative function favoring the tumor’s growth versus serving as immune attractants; perhaps, this balance can somehow be shifted toward effective immune responses.

## Chaperones Outside: Extracellular Chaperones in Immunity

As mentioned above, we have few well-understood mechanisms for the localization of cytosolic chaperones/heat-shock proteins to the cell surface. Similarly, we know little about the release of such chaperones outside the cell (101, 102), despite nearly three decades of research. ER chaperones are already in the secretory pathway, so bypassing KDEL receptors could explain that release. Another mechanism from the cytosol or the ER could involve vesicular release via endolysosomes (103, 104) or by extracellular vesicles (exosomes, microvesicles) (15, 105). While there may be a number of functional roles for extracellular chaperones such as extracellular signaling (106), chaperoning extracellular matrix components (107, 108), and general cytoprotection during injury (109) or in proteostasis (110), much of the research on extracellular chaperones concerns their roles in immunity.

We noted above that the immune properties of extracellular chaperones are intrinsically related to those proteins acting as danger signals when they interact with innate immune cells (111, 112). This stimulatory capacity at a distance is reminiscent of cytokines, and thus led to the term “chaperokine” (113), with particular involvement of cellular TLRs. Extracellular chaperones such as HSP27 (114), HSP60 (115), HSP70 (14), GRP94 (116), HSP90, and GRP170 (117) have all been shown to bind TLRs. Other chaperone receptors include molecules such as CD14, CD36, CD40, LOX1, scavenger receptors SR-A and SREC-1, and CD91 (also called LRP1 and A2MR, the α2-macroglobulin receptor) (118). Thus, innate immune cells, APCs, and a number of other cell types possess receptors implicated in binding extracellular chaperones presumably released by cells under stressful circumstances.

In the area of cancer immunotherapy, the concept of cancer cells producing and releasing chaperones as a form of “auto-vaccination” is an attractive one, and there have been a number of attempts to generate tumor cell lines producing secretable versions of immunogenic chaperones. An example of this is the ER resident vaccine candidate GRP170 (also called ORP150) (22), which has demonstrated danger signal capacity if secreted outside the cell (45) and has been shown to chaperone whole proteins in that secretable form that are antigenic (119). This links the innate immune stimulation by chaperone proteins with the adaptive (targeted) response and demonstrates how chaperones released by or derived from pathogenic tissues may possess both adjuvant and antigen. Numerous other chaperones have been engineered or designed for secretion from tumor cells [reviewed here (120)], including GRP78, which was previously regarded as ineffective as a cancer vaccine (121). The use of an allogeneic tumor cell vaccine with secretable GRP94 (AD100-gp96-Ig) in clinical trials has been reported (122). Putative benefits of this latter form of a vaccine include the “off-the-shelf” utility (i.e., the vaccine may be used on essentially any patient and does not need to come from autologous tumor), the “host versus graft” immune cross-reactivity with the allogeneic cells, and the “continuous-release format” of the GRP94 as an advantage in stimulating immune responses in contrast to the bolus effect from an injectable vaccine. One disadvantage would be the lack of true personal, individual patient tumor-specific antigens available from an autologous preparation, and the constant need for reassurance that the tumor cells were not proliferating.

The presence of chaperones in the extracellular milieu, by intent, or by stress, or damage, offers insight into the biology of the sensation of danger by the immune system, as well as potential practical applications from a vaccine perspective. Our next section will discuss chaperone protein-based cancer vaccines, with an emphasis on ER proteins as components of those vaccines.

## Chaperones as Vaccines: Lone Wolves and Getting the Wagons in a CRCL

The release of chaperones extracellularly, whether by bio-engineering, induced stress, or immune-noticeable forms of cell death, may be reenacted in vaccine scenarios where chaperones are purposefully extracted from tumor cells/tissues and re-introduced to patients, typically by parenteral administration. Benefits in this situation include known dosages, ability to monitor local reactions [e.g., delayed-type hypersensitivity (DTH) responses], and the ability to directly enhance APC activation and migration with topical applicants such as imiquimod (123). Depending on the type of vaccine generated, if the source is a tumor sample, that may become the limiting reagent (124, 125), the heterogeneity of tumors may make accurate “dosing” (i.e., how much of the chaperone is actually from the tumor) more difficult. Nonetheless, patient tumor-derived GRP94/gp96 as an autologous therapeutic vaccine has progressed the farthest in various clinical trials, starting in 2000 [reviewed here (126)] and has since included trials for patients with colorectal and pancreatic cancers, melanoma, non-Hodgkin’s lymphoma, renal cell carcinoma, and continues with Phase II trials for patients with high grade gliomas. The product is currently owned by Agenus^1^, and has gone by HSPPC-96, Oncophage, Vitespen, and currently, Prophage. A major attractive feature has been the low incidence of deleterious side effects, and it has received regulatory approval in Russia for patients at intermediate risk for disease recurrence of renal cell carcinoma, the first such cancer vaccine approved anywhere (127). However, further European Union approval was unsuccessful, where the agency cited, among other issues, a lack of identified antigenic peptides associated with the vaccine preparations^2^. Other heat-shock protein vaccines are also at clinical trial stages (e.g., the HSP110–gp100 complex, NCT01744171)^3^, including one that induces HSPs by inflicting cryoablation or radiofrequency ablation on tumors (NCT00568763) rather than the direct use of individual HSPs as vaccines.

A question that frequently arises in these cancer vaccine scenarios regards the generation of autoimmunity. To some extent, that is indeed the goal of cancer immunotherapy, targeting a tissue that is largely “self.” However, the immune suppressive activities of most cancers likely prevent anti-tumor activity as well as true autoimmune activity. Current immune “checkpoint inhibitors” such as antibodies against CTLA-4 (ipilimumab) that prevent T-cell repression have driven potent anti-tumor responses, but also occasional significant autoimmunity (128). However, such autoimmune responses have not been noted in chaperone-based anti-cancer vaccines (129, 130), but as combination therapies will start utilizing such checkpoint inhibitors (131), vigilance will be essential.

The concept of a multiple-chaperone vaccine arose from the thought that dying cells release entire cohorts of proteins rather than purified batches of individual ones. CRCL is such a multi-chaperone vaccine preparation that initially was shown to contain the four known immunogenic chaperones of that time: HSP70, HSP90, GRP94, and calreticulin (132). The rationale was that these four chaperones, from the cytoplasmic and ER compartments, would likely contain a broader repertoire of tumor antigens from an autologous source, and possibly provide greater APC stimulation than single chaperone vaccines. Rather than purification, CRCL preparation utilized a free solution-isoelectric focusing (FS-IEF) technique that resulted in a large, highly cohesive complex of hundreds of proteins (97, 133). This complex activated DCs yielding high expression of CD40 and MHC I and release of IL-12, resulting in highly stimulated T cells (134, 135). This APC stimulation remained effective even in the face of regulatory T cells (Tregs) (136). Additional studies showed that CRCL-stimulated APCs upregulated CD70, NFκB, and iNOS, along with NO, TNFα, and RANTES production, and enhanced phosphorylation of STAT1 and STAT5, and activation of the AKT and MAPK pathways (137). Depletion of chaperones diminished CRCL’s immune properties (134). Immunological testing demonstrated the presence of the BCR–ABL fusion peptide in CRCL derived from BCR–ABL positive tumors that resulted in peptide-specific responding T cells (138), and further biochemical and proteomic work identified nearly 60 peptides associated with CRCL (139). When used pre-clinically as a single agent in prophylactic and therapeutic vaccination schemes, or as an antigen source for DC vaccines, or in combination with other treatment regimens, CRCL was found effective against numerous murine hematologic malignancies, and against melanoma, fibrosarcoma, breast cancer, and brain tumor models (15, 97, 134, 140–144). CRCL was also shown to drive NK cell pro-inflammatory cytokine and chemokine release (145) as well as bioactive anti-tumor antibody production (143). CRCL, combined initially with the topical TLR stimulant imiquimod, was used as the sole post-surgical therapeutic agent to treat an aggressive metastatic lung cancer in a canine patient (146); the dog’s prognosis was <4 weeks survival, but she survived for 11 months with CRCL treatment. Finally, CRCL is a component of an immunotherapy regimen currently in clinical trials (NCT01998542, NCT01995227).

**Figure 1:**
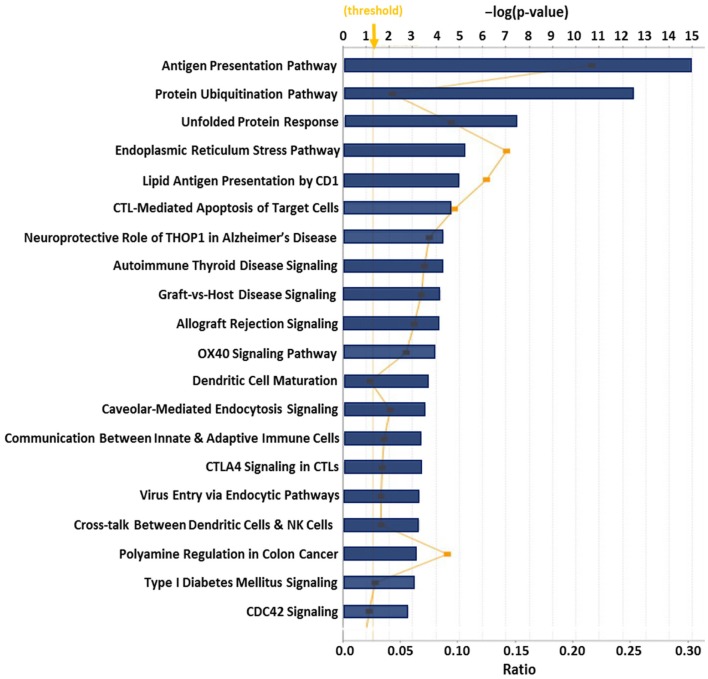
**Top 20 canonical pathways derived from ingenuity pathway analysis (IPA) gene ontology algorithms for the 36 ER and ER-associated proteins from Table 2**. These pathways emerged following IPA “Core Analysis.” Graph shows category scores; “threshold” indicates the minimum significance level [scored as −log(*p*-value) from Fisher’s exact test, set here to 1.25]. “Ratio” (differential yellow line and markers) refers to the number of molecules from the dataset that map to the pathway listed divided by the total number of molecules that map to the canonical pathway from within the IPA knowledgebase.

Previous proteomic work to better biochemically characterize human CRCL preparations from various tumor types (133) identified at least 10 known ER chaperones; re-evaluating all of the data in that publication provided us with 36 proteins that are from the ER or have close associations with that organelle, such as proteasome components (Table 2). Gene ontology (GO) assessment of those proteins using Ingenuity Pathway Analysis (IPA) revealed canonical pathways with clear immunological relevance, including antigen presentation, dendritic cell maturation and communications, and T-cell signaling; the top 20 significantly scoring Pathways are shown in Figure 1. There is also a high overlap among two-thirds of the pathways (not shown).

**Table 2:** **ER/ER-associated proteins identified in a previous proteomic study of human CRCL**.

ID	Symbol	Entrez gene name
Q6DD88	ATL3	Atlastin gtpase 3
P27797	CALR	Calreticulin
P27824	CANX	Calnexin
O14735	CDIPT	CDP-diacylglycerol – inositol 3-phosphatidyltransferase
Q9UKY3	CES1P1	Carboxylesterase 1 pseudogene 1
Q99653	CHP1	Calcineurin-like EF-hand protein 1
Q9BUN8	DERL1	Derlin 1
Q7Z2K6	ERMP1	Endoplasmic reticulum metallopeptidase 1
P30040	ERP29	Endoplasmic reticulum protein 29
Q9BS26	ERP44	Endoplasmic reticulum protein 44
P30443	HLA-A	Major histocompatibility complex, class I, A
D3U3L9	HLA-B	Major histocompatibility complex, class I, B
A5D8 × 1	HLA-C	Major histocompatibility complex, class I, C
P14625	HSP90B1	Heat-shock protein 90 kDa beta (grp94), member 1
P11021	HSPA5	Heat-shock 70 kDa protein 5 (glucose-regulated protein, 78 kDa)
Q9Y4L1	HYOU1	Hypoxia upregulated 1
P13674	P4HA1	Prolyl 4-hydroxylase, alpha polypeptide I
P07237	P4HB	Prolyl 4-hydroxylase, beta polypeptide
O75340	PDCD6	Programed cell death 6
P30101	PDIA3	Protein disulfide isomerase family A, member 3
P13667	PDIA4	Protein disulfide isomerase family A, member 4
Q15084	PDIA6	Protein disulfide isomerase family A, member 6
O60240	PLIN1	Perilipin 1
Q96Q06	PLIN4	Perilipin 4
P28066	PSMA5	Proteasome (prosome, macropain) subunit, alpha type, 5
Q99436	PSMB7	Proteasome (prosome, macropain) subunit, beta type, 7
Q06323	PSME1	Proteasome (prosome, macropain) activator subunit 1 (pa28 alpha)
Q9UL46	PSME2	Proteasome (prosome, macropain) activator subunit 2 (pa28 beta)
O75396	SEC22B	SEC22 vesicle trafficking protein homolog B
Q15437	SEC23B	Sec23 homolog B (*S. cerevisiae*)
P61619	SEC61A1	Sec61 alpha 1 subunit (*S. cerevisiae*)
Q03518	TAP1	Transporter 1, ATP-binding cassette, sub-family B (MDR/TAP)
Q03519	TAP2	Transporter 2, ATP-binding cassette, sub-family B (MDR/TAP)
Q04323	UBXN1	UBX domain protein 1
P09936	UCHL1	Ubiquitin carboxyl-terminal esterase L1 (ubiquitin thiolesterase)
P55072	VCP	Valosin containing protein

*ER and ER-associated proteins identified in a previous proteomic study of human CRCL. Proteins were identified by gel separation, excision, digestion, and mass spectrometry. These proteins were originally found in separate locations in this publication (133), but were extracted and organized into this table*.

**Figure 2:**
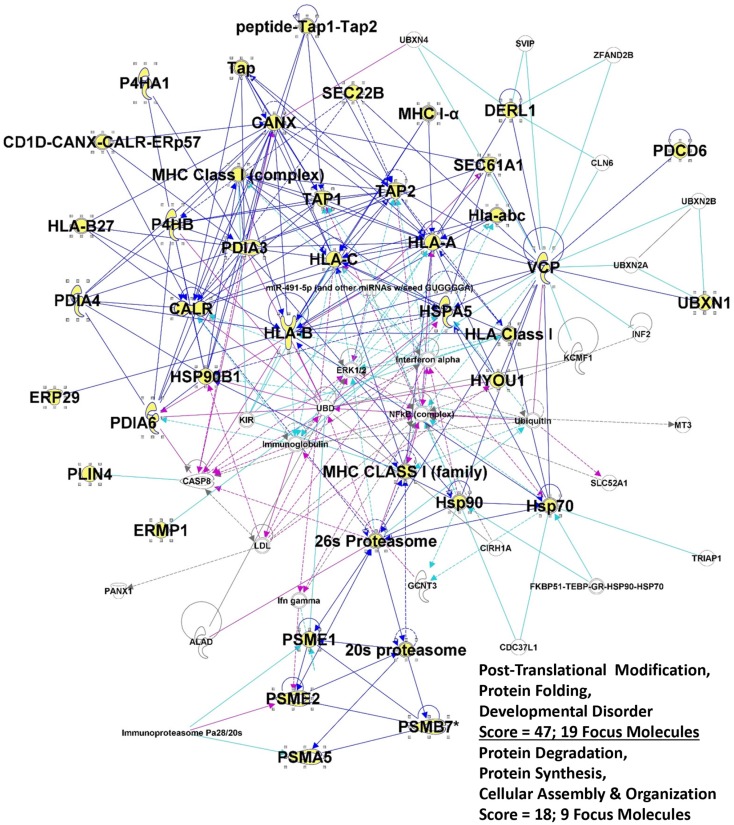
**Intersection of the top 2 IPA interactomes derived from the top networks/associated functions for the 36 proteins listed in Table 2**. Proteins from table are shown in large bold font, and the protein symbols are shown in yellow fill. “Edges” (lines) show connections between or among molecules; solid lines indicated known direct interactions. Dotted lines indicated indirect interactions. Dark blue lines connect proteins from within the entry group; turquoise lines connect proteins that were in the network but not found in our proteomic study. Cranberry colored lines show the intersection of proteins between the two interactomes. “Score” refers to the −log(*p*-value) from Fisher’s exact score, and “Focus Molecules” are “seeds” for generation of focal points or nodes within the network.

One striking outcome from previous IPA applications was a networks/associated functions interactome generated that showed connectivity among various chaperones (both ER and cytosolic), immune-related molecules, nuclear factors, and metabolic enzymes (133). Focusing here on the ER components and associated proteins, we have generated a similar interactome by combining two networks with very high scores (derived from Fisher’s exact test) that seemingly recapitulate the antigen processing pathway for MHC Class I molecules, as well as portions of the ERAD pathways (Figure 2). The selective entries of ER and ER-related proteins may serve to skew the readouts from IPA, but it also suggests that the ER contributions to these interactomes in particular may play heavily into CRCL functionality.

Of the molecules included in this list but not discussed previously (133) from an immune perspective, DERL1 is a member of the ER quality control/ERAD system, where it mediates MHC degradation (147). It is also upregulated in tumors and in epithelial cells exposed to tumors, where it may be involved in angiogenesis (148). Its presence on tumor cell surfaces makes it amenable to antibody targeting (149). ERP44, a PDI family member, plays a controlling role in IgM assembly in B cells (150). The perilipins play roles in the formation and transport of lipid bodies/lipid droplets such as those formed in leukocyte inflammatory responses (151); such lipid bodies are involved in phagocytosed antigen cross-presentation in DCs (152). SEC22B, a SNARE (soluble *N*-ethylmaleimide sensitive fusion attachment protein receptor) protein, is another molecule clearly involved in antigen cross-presentation via maturation of phagosomes (153). SEC23B, while having no clear immune function, is a required gene for cells with high secretory outputs (154), and not surprisingly is overexpressed in hepatocellular carcinomas (155), and perhaps could be regarded as an immune target. UCHL1, also called protein gene product 9.5 (PGP9.5) has been identified as an autoantigen in lung cancer patients (156). Thus, CRCL may contain ER proteins besides the chaperones that may play roles in immune cells or may act as targets of immune responses.

The extraordinary connectivity found in the interactome of these proteins (Figure 2) suggests that there may be structural relationships involved, and indeed bizarre structures were seen in electron microscopy, and large particles were identified by nanoparticle tracking analysis, in the aforementioned publication (133). Prior to that, CRCL was shown to exist biochemically as a large entity of virus-sized proportions by size-exclusion chromatography (97). Which proteins are involved, and what roles they may play, are currently matters of speculation, but conceptually a model for a “relay line” of chaperones sequentially transferring peptides during antigen processing and presentation has been proposed (157). There has even been validation of the peptide transfer (158, 159), suggesting that at least close physical proximity, if not protein–protein contact, is necessary. Nanoparticles for immune stimulation, such as pathogen-like particles, are gaining headway in vaccine research (160, 161). Perhaps, CRCL inadvertently retains some form of particulate assembly due to its cytoskeletal content, and carries antigens within this “cage” due to its chaperone content. The calreticulin component of CRCL may be an especially potent “eat me” signal for APCs, which then view CRCL as an object with viral-like physical properties, and upon engulfment, have endocytosed numerous antigens via the chaperones, including antigens carried by the ER chaperones abundant in CRCL.

Our “peptidomics” work with CRCL-associated peptides implied that the protein origins of those putative antigens came from all cellular compartments, and were high-value targets for immune responses (139). Coupled with the proteomics work mentioned here (133), the GO analyses provide a basis for a systems biology approach to understanding the biochemical (and perhaps structural) mechanisms for the success of the vaccine. The intrinsic roles the ER-derived and -associated components of CRCL are undoubtedly critical to the vaccine’s utility. Further research is required to truly understand the biophysical structure of the vaccine and to determine what impact that has on the immunological responses driven by the vaccine. The ER proteins, representing the connection between the antigen-generating cytosol, the antigen-presenting cell surface, and danger signal activities extracellularly, are undoubtedly vital to the inherent adjuvant/antigen formulation that is CRCL.

## Conflict of Interest Statement

The authors declare that the research was conducted in the absence of any commercial or financial relationships that could be construed as a potential conflict of interest.

## Footnotes

^1^http://www.agenusbio.com/

^2^http://www.ema.europa.eu/docs/en_GB/document_library/Application_withdrawal_assessment_report/2010/03/WC500075459.pdf

^3^www.clinicaltrials.gov
